# Paralemmin-1 is over-expressed in estrogen-receptor positive breast cancers

**DOI:** 10.1186/1475-2867-12-17

**Published:** 2012-05-10

**Authors:** Casey M Turk, Katerina D Fagan-Solis, Kristin E Williams, Joseph M Gozgit, Sallie Smith-Schneider, Sharon A Marconi, Christopher N Otis, Giovanna M Crisi, Douglas L Anderton, Manfred W Kilimann, Kathleen F Arcaro

**Affiliations:** 1 University of Massachusetts, 637 North Pleasant Street, Amherst, MA 01003-9298, USA; 2 Pioneer Valley Life Sciences Institute, 3601 Main Street, Springfield, MA 01107, USA; 3 Department of Pathology, Baystate Medical Center, Springfield, MA 01199, USA; 4 Department of Neuroscience, Uppsala University Biomedical Center, Uppsala S-75124, Sweden

**Keywords:** Paralemmin-1, Breast cancer, PALM, Estrogen receptor, Tissue microarrays, Splice variants

## Abstract

**Background:**

Paralemmin-1 is a phosphoprotein lipid-anchored to the cytoplasmic face of membranes where it functions in membrane dynamics, maintenance of cell shape, and process formation. Expression of paralemmin-1 and its major splice variant (Δ exon 8) as well as the extent of posttranslational modifications are tissue- and development-specific. Paralemmin-1 expression in normal breast and breast cancer tissue has not been described previously.

**Results:**

Paralemmin-1 mRNA and protein expression was evaluated in ten breast cell lines, 26 primary tumors, and 10 reduction mammoplasty (RM) tissues using real time RT-PCR. Paralemmin-1 splice variants were assessed in tumor and RM tissues using a series of primers and RT-PCR. Paralemmin-1 protein expression was examined in cell lines using Western Blots and in 31 ductal carcinomas *in situ,* 65 infiltrating ductal carcinomas, and 40 RM tissues using immunohistochemistry. Paralemmin-1 mRNA levels were higher in breast cancers than in RM tissue and estrogen receptor (ER)-positive tumors had higher transcript levels than ER-negative tumors. The Δ exon 8 splice variant was detected more frequently in tumor than in RM tissues. Protein expression was consistent with mRNA results showing higher paralemmin-1 expression in ER-positive tumors.

**Conclusions:**

The differential expression of paralemmin-1 in a subset of breast cancers suggests the existence of variation in membrane dynamics that may be exploited to improve diagnosis or provide a therapeutic target.

## Background

Paralemmin-1 is a phosphoprotein first identified in brain tissue and is thought to play a role in controlling cell shape, plasma membrane dynamics, and cell motility [1,2]*.* Paralemmin-1 is lipid-anchored to the cytosolic side of the plasma membrane through prenylation and di-palmitoylation of the COOH terminal cysteine cluster [2]. Arstikaitis and colleagues [3] identified paralemmin-1 as a regulator of filopodia induction, synapse formation, and dendritic spine maturation. When overexpressed in fibroblasts paralemmin-1 protein induces cellular expansion and process formation [2]. Knockdown of paralemmin-1 reduces filopodia and compromises dendritic spine maturation [3].

Paralemmin-1 is differentially spliced in a tissue-specific and developmentally-regulated manner. Alternative splicing of the eighth exon of paralemmin-1 is the most common of the splice variants [2] and has been shown to play a role in the recruitment of AMPA-type glutamate receptors [3]. The Δ exon 8 splice variant also has been shown to interact with the third intracellular loop of the D3 dopamine receptor in the hippocampus and cerebellum in rat brain as well as in glial and neuronal cell cultures [2-4]. These studies suggest that the Δ exon 8 splice variant may have distinct functions in specific tissues.

In a global gene expression comparison between two breast cancer cell lines, paralemmin-1 was over expressed in the invasive, estrogen-receptor (ER) negative breast cancer cell line (TMX2-28) as compared to the non-invasive ER-positive parent cell line (MCF-7) [5]. Given the function of paralemmin-1 in plasma membrane dynamics and cell motility in fibroblasts, we hypothesized that paralemmin-1 may play a role in the invasive growth that accompanies metastasis of breast cancers. Here we examine the mRNA and protein expression of paralemmin-1 and its splice variants in ER-positive and ER-negative breast cell lines, primary breast tumors and tissue from reductive mammoplasty surgeries. We found paralemmin-1 to be more frequently expressed in breast cancers than in reduction mammoplasty tissues and more highly expressed in ER-positive breast cancer as compared to ER-negative cancers.

## Results

### Paralemmin-1 mRNA and protein expression in breast cell lines

**Figure 1:**
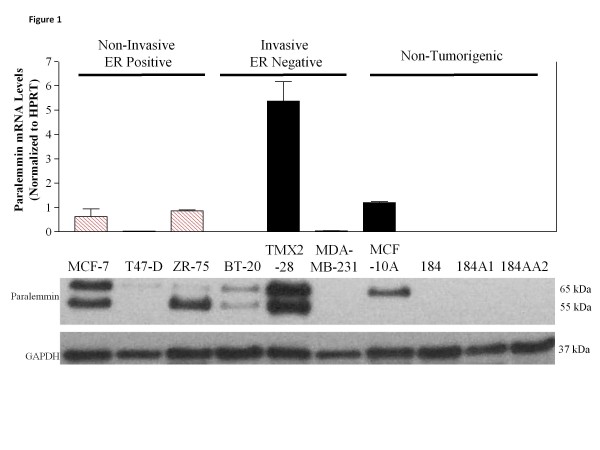
**Paralemmin-1 is differentially expressed in breast epithelial cell lines.** RNA and protein lysates were isolated from tumorigenic and non-tumorigenic breast cell lines. *Top:* Real time qRT-PCR shows mRNA expression of paralemmin-1; means and S.E. from three separate cell cultures are presented; ER-positive cell lines, (hatched bars), ER-negative cell lines (solid bars). *Bottom:* Protein lysates (15 μg) were probed for paralemmin-1 expression by Western immunoblotting. Image is a representative of at least three separate experiments with different biological samples.

A cDNA microarray comparison between the ER-positive MCF-7 breast cancer cell line and its tamoxifen-selected, ER-negative derivative, TMX2-28, had shown that paralemmin-1 mRNA was more highly expressed (8 times greater) in TMX2-28 cells than in MCF-7 cells [5]. Therefore, to investigate the relationship between paralemmin-1 expression and ER status, in the present study we examined the expression of paralemmin-1 in additional breast cancer and non-tumorigenic breast cell lines (Table 1). Real time RT-PCR demonstrated that paralemmin-1 mRNA levels were higher in breast cancer cell lines than in non-tumorigenic breast cell lines (Figure 1 top). Three of the six breast cancer lines examined expressed moderate to high levels of paralemmin-1 mRNA, whereas levels in the three others were low or undetectable. Of the four non-cancer cell lines, only one (MCF-10A) had detectable levels of paralemmin-1 transcript. The higher expression in TMX2-28 as compared to MCF-7 [5] was confirmed. In the whole set of 10 cell lines, however, estrogen receptor status was not a predictor of paralemmin-1 mRNA levels. Among the six cancer cell lines examined, paralemmin-1 mRNA levels were highest in the ER-negative cell line, TMX2-28. However, the next highest expression levels were in two ER-positive cell lines, MCF-7 and ZR-75.

**Table 1:** Breast cell lines examined for paralemmin-1 mRNA and protein expression

**Cell Line**	**Donor Age**	**Pathology**	**ER**	**Paralemmin-1 Expression**	
				**RNA**	**Protein (kDa)**
MCF-7	69	IDC	Pos	medium	65, 55
T47D	54	IDC	Pos	low	65*
ZR-75-1	47	IDC	Pos	medium	65*, 55
BT-20	74	IDC	Neg^1^	low	65, 55
TMX2-28^4^	69	IDC	Neg	high	65, 55
MDA-MB-231	51	Adeno	Neg	low	none
MCF-10A	36	NE	Neg	medium	65
184^2^	15-66	NE	Neg	low	none
184A1^3^	15-66	NE	Neg	low	none
184AA2^3^	15-66	NE	Neg	low	none

^**1**^BT-20 cells express ER mRNA, but do not express ER protein; ^**2**^Non-tumorigenic mammary cell line from Dr. Martha Stamfer’s Laboratory ^**3**^Derivative of 184 cells; ^**4**^Derivative of MCF-7 cells **IDC**: Invasive ductal carcinomas **Adeno**: Adenocarcinoma; **NE:** Normal epithelial; Sources: [6-8]; *indicates a faint band.

Examination of cellular protein by Western Blot analysis using paralemmin-1 antibody revealed differences between breast cancer and non-tumorigenic cell lines (Figure 1 bottom). The protein results were in agreement with the mRNA results: the same cell lines displayed high, moderate, or low expression. Both splice variants of paralemmin-1 (65/55 kDa; +/− exon 8) were detected in variable proportions in the cancer cell lines. Only the upper band was present in the non-tumorigenic MCF-10A cell line. Incubation with the pre-immune control serum revealed no bands at 55 and 65 kDa (data not shown).

### Paralemmin-1 RNA and protein expression in clinical breast tumors and reduction mammoplasty tissue

**Figure 2:**
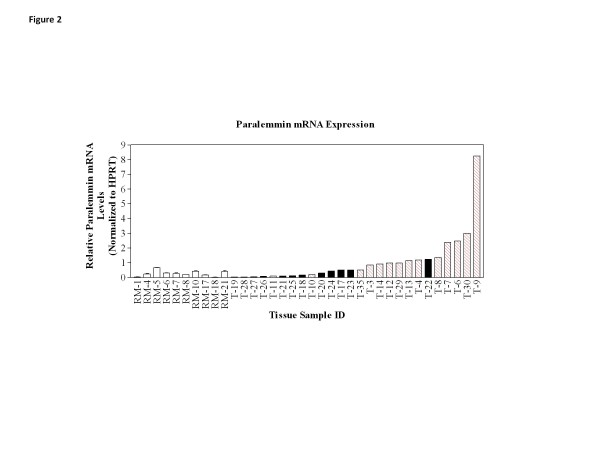
**Paralemmin-1 mRNA is highly expressed in ER-positive human tumor samples.** Gene expression of paralemmin-1 was determined in 26 frozen breast carcinoma tissue (T) and 10 reduction mammoplasty (RM) samples (open bars) using real time RT-PCR. ER-positive tumors were assigned the numbers 1–18 (red hatched bars) while ER-negative tumors were assigned 19–30 (solid bars) and then sorted by paralemmin-1 expression.

Paralemmin-1 mRNA levels were assessed in frozen breast cancer tumor tissue from 26 cases and in 10 reduction mammoplasty specimens. Using real time RT-PCR, relative paralemmin-1 values were found to range over 270 fold; from a low of 0.03 to a high of 8.23 relative units (Figure 2). In contrast to the cell line data, paralemmin-1 expression correlated with ER status in tumors. Benign breast epithelial cells constitutively express focal low levels of hormone receptors. Paralemmin-1 was significantly higher in ER-positive as compared to ER-negative tumors (p < 0.001) and in ER-positive tumors as compared to reduction mammoplasty tissue (p < 0.01). There was no significant difference in paralemmin-1 levels between ER-negative tumors and reduction mammoplasty tissue (p > 0.05).

**Figure 3:**
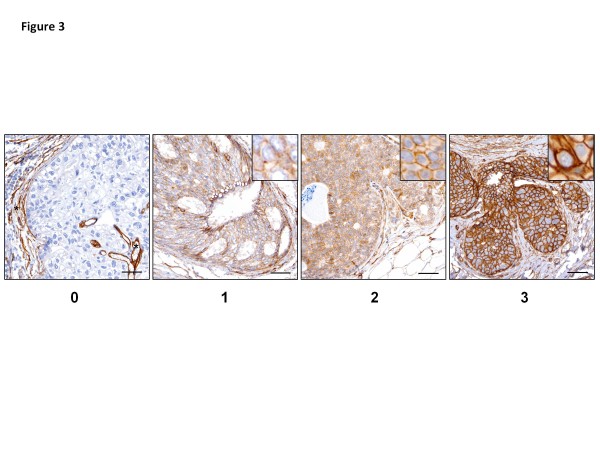
**Scoring system used to evaluate paralemmin-1 expression in TMA from breast tumors and reduction mammoplasty specimens.** Intensity of immunoreactivity of epithelial cells was used to assign scores of 0, 1, 2, and 3. A 0 was little to no staining, a 1 was considered to be “light” intensity of staining; a 2 was considered to be “moderate” intensity of staining, a 3 was considered to be “strong” intensity of staining. Images are from a DCIS TMA with inset in score 1, 2 and 3 images showing increasing intensity of paralemmin-1 plasma membrane staining in tumor cells. Image for score 0 shows negative paralemmin-1 staining in tumor cells and small peritumoral lymphocytes (left). Intratumoral and peritumoral vascular endothelial cells (*) and elongated stromal cells are positive for paralemmin-1. Scale bars 50 micron.

**Figure 4:**
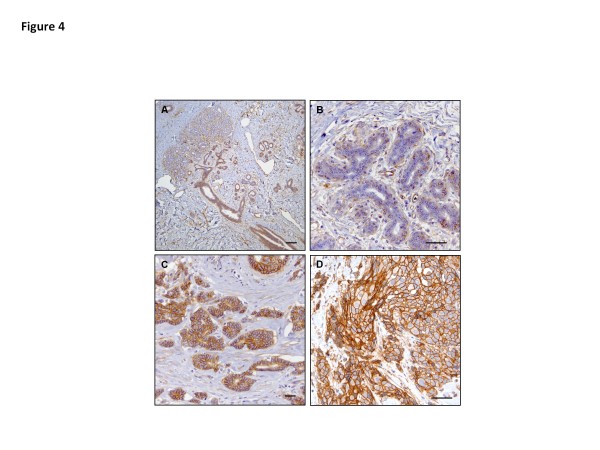
**Paralemmin-1 expression in breast epithelial cells is primarily localized to the cell membrane and is more highly expressed in tumor tissue than reduction mammoplasty.** Breast tumor TMAs and reduction mammoplasty tissues were prepared, stained, and scored as described in the Methods. Representative images of paralemmin-1 immunoreactivity in reduction mammoplasty tissues (A and B, low and high magnification) show partial staining in terminal duct and acinar ductal epithelial cells. Paralemmin-1 is present in stromal cells and endothelial cells (image B *). In contrast, in tumor tissues (C and D, high magnification) significant immunoreactivity is present in the malignant epithelial cells where the staining is localized to the cell membrane. Scale bar image A 100 micron. Scale bars image B, C, D 50 micron.

Paralemmin-1 protein expression was examined immunohistochemically using tissue microarrays (TMA) constructed from formalin-fixed and paraffin embedded (FFPE) tissue of 40 RM, 31 ductal carcinoma *in situ* (DCIS) and 65 invasive ductal carcinoma (IDC) cases. In benign reduction mammoplasty breast tissue paralemmin-1 immunostaining was focally present in glandular epithelial and myoepithelial cells, with variability in intensity (weak to strong) and in percentage of cells staining within each case and between cases. Paralemmin-1 was constitutively expressed in vascular endothelial cells and variably expressed in stromal cells. In this study we focused on evaluating paralemmin-1 staining characteristics of benign epithelial cells in RM and tumor epithelial cells. Figure 3 provides examples of breast cancer samples that received scores of 0, 1, 2, or 3 based on intensity of the stain, and shows paralemmin-1 staining in epithelial tumor cells to be primarily localized to the cell membrane (inserts) (see Methods for details of scoring and analysis). Figure 4 shows paralemmin-1 expression in representative RM (A and B) and IDC (C and D) cases. Roughly 70% of both the DCIS and IDC cases showed strong reactivity to the paralemmin-1 antibody (Table 2). In contrast, only 27% of RM cases were scored as strong, the remainder showed little to no immunoreactivity with the paralemmin-1 antibody. Examination of the relationship between paralemmin-1 staining and hormone receptor status revealed a trend for higher paralemmin-1 expression in tumors that were positive for ER and/or progesterone receptor (PR).

**Table 2:** Paralemmin-1 protein expression in reduction mammoplasty and breast cancer cases

	**RM**	**DCIS**	**IDC**	**DCIS & IDC**					
				**ER**		**PR**		**HER2**	
				**Pos**	**Neg**	**Pos**	**Neg**	**Pos**	**Neg**
**Weak**	29 (73)	7 (23)	20 (31)	21 (26)	8 (57)	16 (23)	12 (48)	7 (29)	20 (30)
**Strong**	11 (27)	24 (77)	45 (65)	60 (74)	6 (43)	54 (77)	13 (52)	17 (71)	47 (70)

Number and (percent) of cases in each category that were scored either weak or strong for paralemmin-1 protein expression. **RM**: reduction mammoplasty; **DCIS**: ductal carcinoma in situ; **IDC**: invasive ductal carcinoma; **ER:** estrogen receptor; **PR**: progesterone receptor; **HER2**: human epidermal growth factor receptor 2.

Greater than 70% of ER and/or PR-positive tumors were scored as having high levels of paralemmin-1. In contrast, in tumors lacking ER and/or PR, paralemmin-1 levels were equally divided between weak and strong. No relationship between human epidermal growth factor receptor (HER2) status and paralemmin-1 levels was detected (Table 2).

### Paralemmin-1 exon-splice variants in clinical breast tumors and reduction mammoplasty tissues

**Figure 5:**
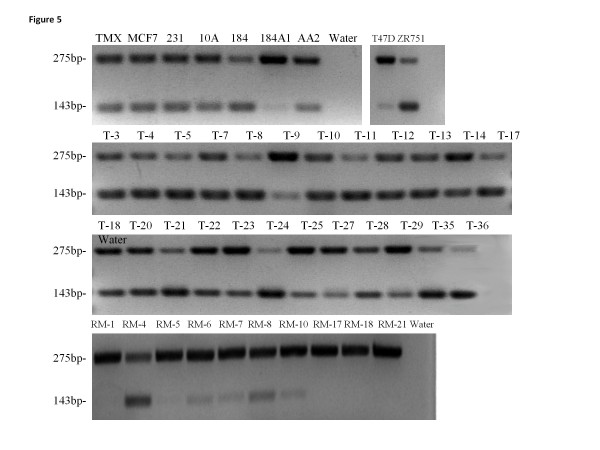
**Tumor tissues and breast cell lines express a higher proportion of the Δ exon 8 splice variant of paralemmin-1 than do reduction mammoplasty tissues.** RNA was isolated and amplified with RT-PCR analysis using a primer set to detect Δ exon 8 splice variant. RT-PCR products were separated on a 2% low melting agarose gel and visualized by ethidium bromide. The full abbreviations of the cell lines are in Table 1. Tumor tissue samples are represented by the prefix T and reduction mammoplasty tissue samples are represented by the prefix R. Numbers on the left of the figures represent the full length product (275 bp) and the Δ exon 8 splice variant (143 bp).

RNA from nine cell lines, and from 24 of the 26 tumor samples and all ten of the RM samples shown in Figure 2 was examined for the presence of paralemmin-1 splice variants; five primer sets designed to detect mRNA missing exons 4, 5, 6, 7, or 8 (Table 3) were used in RT-PCR to determine the number and size of products. Primers for detecting variants missing exons 4, 5, or 7 yielded only full length products in all cell lines, tumor and RM samples examined (data not shown). Primer set 6 yielded full length products in all cell lines, tumor and RM tissues and also resulted in a second smaller product in one tumor, indicating that a single tumor expressed the exon 6-deleted splice variant (data not shown). In contrast, primer set 8 revealed the presence of the exon 8 splice variant. Of the nine cell lines examined, all expressed the full length product and 7 had a robust signal for the splice variant. Similarly, all 24 of the tumor samples expressed both the full length and Δ exon 8 splice variant, whereas in 9 out of 10 RM samples the splice variant was clearly less abundant or undetectable (Figure 5). Note that the cDNA visualized in the gel are the products of 45 PCR cycles. Therefore, while several cell lines and all the RM samples had low levels of paralemmin-1 mRNA as evaluated by real time RT-PCR (shown in Figure 1 top and Figure 2) all of these samples yielded paralemmin-1 PCR products after 45 cycles.

**Table 3:** Primers used to detect exon-deleted splice variants in paralemmin-1

		**Primer Sequence**	**Nucleotide start site**	**Product Lengths (bp)**	
				**Full**	**Deleted**
PS4	sense	AGGCGGAGATCGAGAACAAG	282	221	90
	antisense	CCAGCACCTCAATTTCCTTC	503		
PS5	sense	CAGGACGACGAGCAGAAGA	431	233	82
	antisense	GGAGACTCGCTTGTCTTTGG	664		
PS6	sense	AAGGAAATTGAGGTGCTGGA	485	185	163
	antisense	CGTGTTGGAGACTCGCTTG	670		
PS7	sense	TGATGAATTCACAGCAGACG	612	150	90
	antisense	GTCTCCCCTGTCACCTTGTC	762		
PS8	sense	ACAAGCGAGTCTCCAACACG	651	275	143
	antisense	CCGCTTTGTGGATGAGTTC	926		

## Discussion

Paralemmin-1 is expressed in numerous tissues but is most highly expressed in the brain, where it is thought to affect plasma membrane dynamics, cell shape [2], and ultimately the development and plasticity of the nervous system. It may do so by serving as an adaptor protein that connects membrane proteins with each other, with the cytoskeleton, or with motor proteins [1]. These properties of paralemmin-1 might implicate this protein not only in normal morphogenesis but also abnormal development and cancer. Here we show for the first time that paralemmin-1 is expressed in breast cancer cell lines and human breast cancers.

Our discovery of paralemmin-1 overexpression in breast cancer cell lines came from a cDNA array comparison between the ER-positive breast cancer cell line, MCF-7, and its tamoxifen-selected, ER-negative derivative, TMX2-28. [5]. We proposed that the overexpression of paralemmin-1 may contribute to the invasive nature of the TMX2-28 cells, and possibly to the greater metastatic potential of ER-negative breast cancers. Therefore, we examined paralemmin-1 RNA and protein expression in a larger sample of 10 breast cell lines. Contrary to our expectation, paralemmin-1 expression was not inversely correlated with ER status among the tumorigenic and non-tumorigenic cell lines we examined. Furthermore, when we examined RNA levels in 26 primary breast tumors we found paralemmin-1 expression to be significantly higher in ER-positive as compared to ER-negative tumors. This finding was confirmed when we examined tissue samples by immunohistochemistry; a greater percentage of ER-positive tumors were scored as having ‘high’ levels of paralemmin-1 protein, suggesting that paralemmin-1 expression may still play a role in the differences observed between ER-positive and ER-negative breast tumors. Although it is conceivable that hormonal status may influence the variability of paralemmin-1 expression in benign breast epithelium, we did not evaluate this possible association which is beyond the scope of this study.

Kutzleb and colleagues [1] investigated the cellular and subcellular localization of paralemmin-1 in rat brain and kidneys. Their study revealed that paralemmin-1 in the brain was widely distributed in most neuron cell bodies, axons, dendrites and glial processes, while in the kidney paralemmin-1 showed differential expression based on the cell type with a mosaic of paralemmin-1-positive and -negative cells in the proximal and distal tubules, parietal epithelium of Bowman’s capsule and the endothelium of many blood vessels. At a subcellular level paralemmin-1 has been shown to concentrate at the apical membranes of adrenal chromaffin cells, but at the basolateral membranes of proximal and distal tubule cells in the kidney. Paralemmin-1 detected in the cytoplasm was usually associated with endomembranes.

The present study provides the first histological characterization of paralemmin-1 immunolocalization in normal and cancerous mammary tissue. In the breast paralemmin-1 is constitutively expressed in vascular endothelial cells (lymphatic and small blood vessel), and variably expressed in stromal cells (not further defined). Paralemmin-1 is focally expressed in breast ducts and lobules, showing variable expression in epithelial cells within each case and between cases, from no staining to strong focal staining. The subcellular localization of paralemmin-1 in breast epithelium is consistent with other tissue types: paralemmin-1 was primarily detected at the cell membrane and to a much lesser extent in the cytoplasm. The major difference between paralemmin-1 staining in normal breast tissue (RM) and breast cancer (DCIS and IDC) was the greater frequency of strong staining in cancer tissue.

There have been only a few reports of paralemmin-1 expression in cancer tissue. In two reports paralemmin-1 was identified through mRNA microarray analyses as being upregulated in cancer cells. Paralemmin-1 was upregulated in androgen-independent relative to both androgen-dependent tumors and to normal controls in a mouse prostate model [9], and high levels of paralemmin-1 and paralemmin-2-AKAP2 expression were correlated with an invasive morphological phenotype of breast cancer cell lines [10].

Duncavage and colleagues [11] investigated the expression of paralemmin-2 in non-small cell lung carcinomas because it is a potential target of the microRNA-221, which they showed to be down regulated in nonrecurrent tumors. However, paralemmin-2 was equally expressed in both recurrent and nonrecurrent non-small cell lung carcinomas and it was not determined whether paralemmin-2 levels were higher in tumors than in normal tissue. In the present report we not only found that paralemmin-1 expression was greater in a subset of breast tumors, but also that paralemmin-1 expression was greater in tumor tissues than in RM tissues. We have not examined the expression of benign epithelium adjacent to tumor, however our observations in RM tissue suggest that this protein is expressed at low levels in normal breast tissue.

Phosphorylation and mRNA splicing of paralemmin-1 is tissue-specific, developmentally regulated and contributes to the electrophoretic heterogeneity frequently seen on Western Blots. Amino acids 154–230 of paralemmin-1, which correspond to exon 8 of the mRNA sequence, have been shown to interact with the third intracellular loop of the D3 dopamine receptor in the hippocampus and cerebellum in rat brain, and in glial and neuronal cell cultures [4]. Thus variants in which exon 8 is spliced out of the RNA likely result in specific changes in paralemmin-1 function in different tissue types. In the present study we detected significant expression of paralemmin-1 RNA missing exon 8 in both ER-positive and ER-negative breast cancers. In contrast, there was very little expression of the Δ exon 8 splice variant in the RM tissue. At present we do not know whether normal tissue adjacent to the cancer expresses paralemmin-1 or the Δ exon 8 splice variant. Use of laser-capture microdissection to isolate RNA from tumor and adjacent benign tissue may be valuable in defining the expression and role of paralemmin-1 in breast carcinogenesis.

## Methods

### Cell culture and RNA purification

TMX2-28 cells were kindly provided by Dr. John Gierthy (Wadsworth Center, Albany, NY). MCF-7 and BT-20 cells were purchased from the American Type Culture Collection (Manassas, VA). The cell lines 184, 184A1, and 184AA2 were generous gifts from Dr. Martha Stampfer (Ernest Orlando Lawrence Berkeley National Laboratory, Berkeley, CA). MDA-MB-231, T47D, ZR75, and MCF-10A cells were obtained from the Wadsworth Center (Albany, NY). TMX2-28 and MCF-7 cells were grown in Dulbecco’s modified eagle medium (DMEM; without phenol red) supplemented with 5% calf serum (Hyclone, Logan, UT), 2.0 mmol/L of L-glutamine, 0.1 mmol/L of nonessential amino acids, 10 ng/mL of insulin, 100 units/mL of penicillin, and 100 μg/mL of streptomycin. T47-D, ZR-75, BT-20 and MDA-MB-231 cells were maintained in complete growth medium, which included 10% FBS according to the American Type Culture Collection protocol. MCF-10A were grown in mammary epithelial growth medium (Clonetics, Walkersville, MD) which contains growth factors but no serum, whereas 184, 184A1, and 184AA2 were cultured in mammary epithelial growth medium according to Dr. M. Stampfer’s protocol as posted on her website [12]. Cells were maintained in a humidified incubator at 37°C with 5% CO_2_. RNA was isolated from cell cultures with TRI Reagent (Molecular Research, Cincinnati, OH) according to the manufacturer’s protocol. Isolated RNA was further purified using the Qiagen RNeasy kit with on-column DNase digestion (Qiagen, Valencia, CA).

### Protein isolation and western immunoblotting

Cell cultures were lysed with pre-chilled SDS buffer (1% SDS and .60 mM Tris–HCl, pH 5) and extracts were used for Western immunoblotting. Protein lysates (15 μg) were mixed with NuPage sample buffer and reducing agent (Invitrogen, Carlsbad, CA), heated at 70°C for 10 min and then separated on a 10% Tris–HCl polyacrylamide gel using the Mini-PROTEAN 3 cell (Bio-Rad, Hercules, CA) according to the manufacturer’s protocol. Separated proteins were transferred to an Immun-Blot PVDF membrane using the Mini Trans-Blot electrophoretic transfer cell and protocol (Bio-Rad, Hercules, CA). Membranes were incubated in blocking buffer (5% nonfat dry milk/TBS and 0.1% Tween 20) for 30 min at room temperature with gentle shaking, and then incubated with anti-paralemmin-1 rabbit polyclonal antibody diluted 1:100,000 overnight at 4°C, followed by incubation with the secondary antibody (anti-rabbit IgG linked to horseradish peroxidase; diluted 1:2,000; Cell Signaling Technology, Beverly, MA) for 1 h at room temperature. Chemiluminescent signals were detected with the SuperSignal West Pico kit and protocol (Pierce, Rockford, IL). Membranes were stripped according to manufacturer’s protocol using Restore stripping buffer (Thermo Scientific, Rockford, IL) and reprobed for glyceraldehyde 3-phosphate dehydrogenase (GAPDH ; Cell Signaling Technology; diluted 1:10,000) overnight at 4°C, followed by incubation with the secondary antibody (anti-rabbit IgG linked to horseradish peroxidase; diluted 1:2,000; Santa Cruz Biotechnology, Santa Cruz, CA) for 1 h at room temperature.

### Real-Time RT-PCR

Real-time RT-PCR was performed as previously described [5]. RNA samples were reverse transcribed and amplified using the One-Step RT-PCR kit (Qiagen, Valencia, CA) in the Roche Light Cycler (Roche, Indianapolis, IN). Total RNA (75 ng) was incubated with Qiagen RT-PCR master mix including primers (25 μmol/L each) and SYBR Green I nucleic acid stain (diluted 1:5000; Molecular Probes, Eugene, OR) in pre-cooled capillaries (Roche, Indianapolis, IN) and was reverse transcribed. Following reverse transcription, samples were heated, to activate the HotStar Taq DNA polymerase and to simultaneously inactivate the reverse transcriptase. The generation of amplified products was monitored over 45 PCR cycles by fluorescence of intercalating SYBR Green. Relative mRNA levels were normalized to hypoxanthine ribosyltransferase (HPRT) levels to control for RNA quality and concentration. Gene-specific primers were designed using Primer3 [13]: HPRT NM_000194: ACCCCACGAAGTGTTGGATA (nucleotide 587, sense), AAGCAGATGGCCACAGAACT (nucleotide 834, antisense); Paralemmin-1 NM_002579: GAGTGAGCCACTCCTTGTCC (nucleotide 2057, sense), GTGCTCCAAGCCCAGTAGAG (nucleotide 2241, antisense).

### Human tissue

Institutional Review Board approval was obtained from Baystate Medical Center, Springfield, MA, and all samples, both frozen and fixed were identified numerically to maintain patient anonymity. For tumor tissues, grade, ER, PR, and HER2 status were recorded at the time the sample was collected.

#### RNA analysis

Twenty-six frozen breast tumor samples were retrieved from Baystate Medical Center, Department of Surgical Pathology, sectioned to ≤0.5 cm in thickness and immediately placed in pre-chilled RNA Later-Ice (Ambion, Austin, TX) for 24 hours, at which time tumor samples (20–30 mg) were homogenized and RNA isolated using the Qiagen RNeasy Fibrous Tissue kit. Ten fresh RM samples were collected at surgery (Baystate Medical Center) and snap frozen. RNA was isolated from RM using RNeasy mini columns (Qiagen, Valencia, CA) followed by a cleanup with Turbo DNA-*free* (Ambion, Austin TX).

#### Tissue microarrays

Ninety-one FFPE primary breast carcinomas (31 DCIS and 65 IDC) and 40 FFPE RM tissue samples were retrieved from Baystate Medical Center, Department of Pathology.

### Tissue microarrays immunohistochemistry

Tissue microarrays (TMAs) were constructed by extracting three 1.0-mm diameter cores from each FFPE tissue block and re-embedding them into a recipient paraffin block containing holes spaced 2.0 mm apart. A total of five TMAs were prepared containing tissue from 40 RM cases (two TMAs), 31 DCIS cases (one TMA), and 65 IDC cases (two TMAs). Four-micron tissue sections were placed on charged slides, deparaffinized in xylene, and rehydrated in graded ethanol solutions. Slides were rinsed in water and incubated in Citra Plus Buffer (BioGenex, San Ramon, CA) under the following conditions for antigen retrieval: microwave for 3 min, cool for 1 min, heat at 98°C for 10 min, and cool for 20 min. Staining was performed on a Dako Autostainer using anti-paralemmin-1 rabbit antibody diluted 1:5000 [1] and Dako Envision Plus labeled polymer horseradish peroxidase reagents. Two anatomic pathologists independently and blindly scored a slide from each TMA for paralemmin-1 immunoreactivity, assigning values of 0 through 3 for the intensity of staining and descriptors of focal (low) or diffuse (high) or variable for the percentage of tumor cells stained. After at least three days elapsed, each pathologist, without access to their previous scores, examined and scored a second slide from each of the five TMAs. For each case, the highest score given to any of the three punches was used for analysis. The intensity scores of 0 and 1 were classified as ‘weak staining’ and scores of 2 and 3 were classified as ‘strong staining’.

### RT-PCR and analysis of exon splice variants

RNA samples from breast cell lines, frozen breast tumors and RM tissue were amplified as described above except the SYBR Green was substituted with RNase-free water. The PCR products were separated by electrophoresis on a 2.0% low melting point agarose gel and visualized with ethidium bromide. Exon-specific primers for paralemmin-1 (Table 3) were designed using Primer3, and checked for extendible primer dimers using PerlPrimer [14]. Each set was expected to yield either one or two bands. Two bands indicate the expression of both the full length and exon deleted products; one band indicates expression of only the full length (larger) or exon-deleted (smaller) product.

### Statistical analyses

Data in Figures 1 and 2 were analyzed and graphed with GraphPad Prism version 3.02 (GraphPad Software, Inc., San Diego, CA). Mann–Whitney U tests (significance set at P < 0.05) were used to compare ER status and paralemmin-1 expression in breast tumors, and to compare paralemmin-1 levels between RM and tumors.

## Abbreviations

DCIS: Ductal carcinoma *in situ*; ER: Estrogen receptor; FFPE: Formalin fixed paraffin embedded; HER2: Human epidermal growth factor receptor 2; IDC: Invasive ductal carcinoma; PR: Progesterone receptor; RM: Reduction mammoplasty; TMA: Tissue microarray.

## Competing interests

The authors declare that they have no competing interests.

## Authors’ contributions

CMT, KDF-S, KEW and JMG contributed to the cell culture analyses, both protein and mRNA, and to the mRNA analyses in primary tumor and RM. CMT designed the primers for the splice variant analysis; SSS provided guidance and RM tissue; SAM performed the immunohistochemistry; CNO supervised optimization of the performance of the paralemmin-1 antibody, and scored all TMAs; GMC reviewed cases, selected tissue blocks to be used for the TMAs, and scored all TMAs; DLA provided assistance in design and statistical analysis; MWK provided the paralemmin-1 antibody and interpretation of results; KFA provided overall guidance, analysis and prepared the manuscript. All authors read, critically evaluated, edited, and approved the final manuscript.
